# A unique case of glaucoma associated with heterotopic bone formation in the anterior chamber angle

**DOI:** 10.1016/j.ajoc.2023.101959

**Published:** 2023-11-10

**Authors:** Rodolfo Bonatti, Bonnie He, Sylvia Pasternak, Brennan D. Eadie

**Affiliations:** a Faculty of Medicine, Dalhousie University, Halifax, Nova Scotia, Canada; b Department of Ophthalmology and Visual Sciences, Dalhousie University, Halifax, Nova Scotia, Canada; c Department of Pathology and Laboratory Medicine, Dalhousie University, Halifax, Nova Scotia, Canada; d Department of Medicine, Division of Dermatology, Dalhousie University, Halifax, Nova Scotia, Canada

**Keywords:** Glaucoma, Open angle, Bone, Anterior chamber angle, Trabeculectomy

## Abstract

**Purpose:**

To describe a unique case of unilateral open angle glaucoma secondary to heterotopic bone formation in the anterior chamber angle.

**Observations:**

A 57 year-old male with an unremarkable history presented with right eye pain. Anterior segment examination demonstrated a solid, white deposit overlying the trabecular meshwork and peripheral iris associated with an intraocular pressure of 44 mmHg. The left eye examination was unremarkable. Biopsy of the material surprisingly showed heterotopic bone. Removal of the material and medical treatment were unable to adequately control the intraocular pressure and a trabeculectomy was successfully performed.

**Conclusions and Importance:**

This case demonstrates a unique cause of secondary open angle glaucoma: heterotopic bone formation in the anterior chamber angle.

## 1 Introduction

Secondary glaucomas occur when an identifiable abnormality has a role in the pathology. Heterotopic ossification is the formation of mature bone over other types of soft tissue, generally associated with trauma and surgery.^1^ In the eye, the literature describes heterotopic ossification of the retina, choroid and orbital tissues,^2^ but there is no description of glaucoma caused by bone tissue formation. We report a case of heterotopic bone formation over the trabecular meshwork elevating intraocular pressure. We present the initial biomicroscopic appearance with ultrasound biomicroscopy (UBM) image, surgical biopsy with pathology slides and treatment of glaucoma.

## 2 Case report

A 57-year-old male presented to the Ophthalmology Emergency Clinic at Dalhousie University complaining of redness and pain in his right eye that had been present for several weeks. The patient denied a history of trauma, fever, or respiratory problems and was otherwise healthy. The patient's visual acuity was 20/20 in both eyes, with intraocular pressure (IOP) measuring 44 mmHg OD and 16 mmHg OS.

**Fig. 1:**
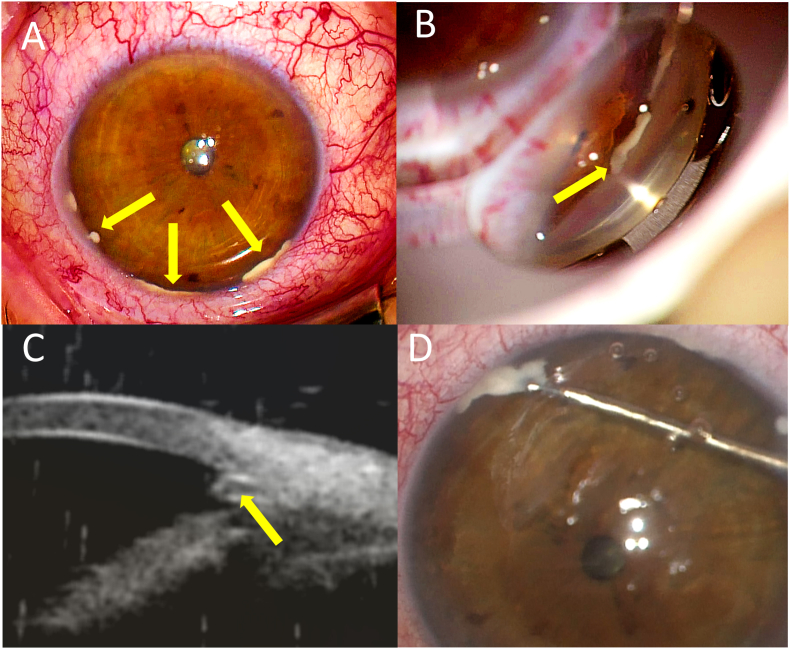
(A) Anterior view of the patient's right eye, showing inferior and temporal deposits of white material (yellow arrows). (B) Gonioscopic view of the materials in the anterior chamber angle. (C) Ultrasound biomicroscopy image highlighting the position of the deposits over the anterior chamber angle. (D) Surgical removal of the deposit with microforceps. (For interpretation of the references to colour in this figure legend, the reader is referred to the Web version of this article.)

Upon examination, the patient's right eye showed mild ocular hyperemia with a white material deposited over the peripheral iris (Fig. 1A). The anterior chamber was quiet. Gonioscopy revealed clusters of white deposits overlying the trabecular meshwork (Fig. 1B). The anterior slit lamp examination of the left eye was normal, and both eyes' fundus examination were grossly within normal limits with the exception of optic disc cupping. There was evidence of a diffuse superior arcuate scotoma with nasal step in the right eye on Humphrey automated visual field, and optical coherence tomography results were consistent with moderate glaucomatous optic neuropathy of the right eye. The visual field and OCT of the left eye were both normal.

Ultrasonic biomicroscopy (UBM) examination confirmed the deposition of the material over the trabecular meshwork (Fig. 1C). The patient was referred to the glaucoma service for further evaluation and management. He was treated with aqueous suppressants, resulting in a mild decrease in IOP to 27 mmHg. The deposits remained unchanged during treatment. We performed a biopsy of the anterior chamber material to rule out ocular lymphoma or masquerade syndrome. The surgery was uncomplicated, and using a direct goniolens and microforceps (Fig. 1D), we removed the majority of the hard, white material from the anterior chamber angle.

**Fig. 2:**
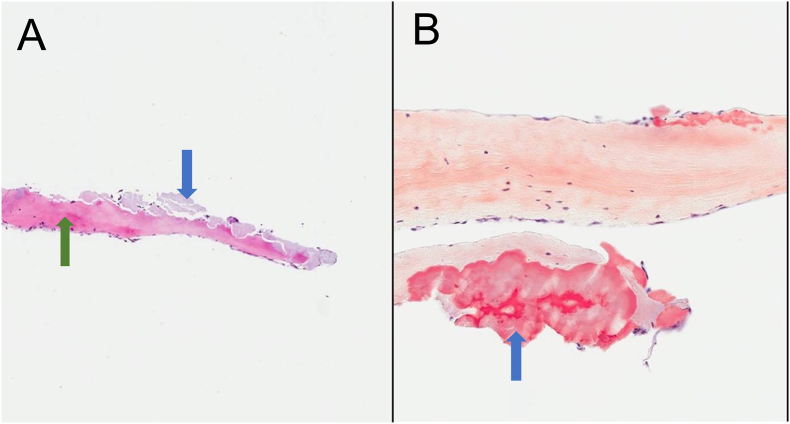
(A) Hematoxylin and eosin stain showing the heterotopic bone (green arrow) and amyloid (blue arrow) deposits. (B) Congo red stain shows bright red positivity in areas of amyloid deposition (blue arrow). (For interpretation of the references to colour in this figure legend, the reader is referred to the Web version of this article.)

Microscopic examation of the removed material did not show lymphocytic proliferation or fungal infection. The material consisted of heterotopic bone and amorphous eosinophilic material with staining properties of amyloid (Congo Red positivity and apple greenbirefrongence under polarized light) (Fig. 2A and B). The material was sent to the Mayo Clinic, confirming the presence of Congo Red positive material. However, no amyloid precursor protein was identified using mass spectrometry. Electron microscopic examination revealed the presence of fibrils that were longer than the 8–12 nm characteristic of amyloid fibrils.

Despite maximum tolerated medical therapy and removal of the heterotopic bone from the right eye, the patient's intraocular pressure remained elevated (27–32 mmHg on multiple visits). We therefore performed a trabeculectomy with mitomycin-C to address the elevated pressure. One month after the surgery, the patient's intraocular pressure was 10 mmHg. We continue to observe this patient.

## 3 Discussion

Ectopic ossification is defined as the formation of extraskeletal bone tissue at the level of soft, richly vascularized tissues.^3^ It is structured bone tissue controlled by osteoblasts and osteoclasts.^4^ Intraocular ossification usually occurs in association with chronic eye diseases (*e.g.,* phthisis bulbi, chronic retinal detachment, chronic inflammation, microphthalmia, buphthalmos, age-related macular degeneration, and trauma).^2^^,^^5^ Our patient did not report any previous trauma or ocular conditions. This case represents a unique presentation of idiopathic anterior chamber angle ossification in human, and we describe this in association with glaucoma.

The mechanisms of ectopic bone formation in the eye are complex and not entirely understood. There is consensus that the pathogenesis involves chronic inflammation, trauma, drusen, and differentiation of mesenchymal stem cells.^2^ Two main types of osteogenic precursor cells can induce ossification: (i) determined precursor cells present in the bone marrow stroma, and (ii) inducible precursor cells, which circulate in the blood and connective tissue.^6^ Inducible osteogenic precursor cells require an agent, such as retinal pigment epithelium (RPE) cells, to induce bone formation. Pluripotent RPE cells may undergo mesenchymal differentiation to different phenotypes, including fibroblasts and osteoblasts.^7^ Multifunctional cytokines from RPE cells (*e.g.*, GDF-5, BMP-7, TGF β1) appear to play an essential role in ectopic bone formation in the eye.^6^^,^^8^

Interestingly, idiopathic bone formation in the anterior segment is a condition that has previously been observed in guinea pigs. One study reported a prevalence of 0.8 % for heterotopic bone formation in guinea pigs at the anterior ciliary body.^9^ Besides the inflammatory theory, the authors hypothesize a link between this condition and the supplementation of ascorbic acid (Vitamin C), in which high levels concentrated in the aqueous humour could promote localized mineralization and bone formation in the ciliary body.^9^ Although Vitamin C supplementation increases its concentration in the anterior chamber,^10^ no further studies have been done to support this hypothesis. Our patient did not endorse an excess of Vitamin C supplementation.

In human eyes with glaucoma, a three-fold increase in activity of the calcification marker alkaline phosphatase has been observed in trabecular meshwork tissue,^11^ suggesting that abnormalities in the trabecular meshwork of patients with glaucoma could predispose to heterotopic bone formation. Also, interestingly, it has been hypothesized that steroids can induce calcification leading to glaucoma; however, our patient did not report using any steroids leading up to his diagnosis.^11^^,^^12^ Finally, since the extent of bone formation in our patient may not have been sufficient to cause an IOP of 44 mmHg solely by means of a trabecular meshwork blockade, it is possible that the patient had an unknown preexisting glaucomatous condition leading to heterotopic bone formation prior to him presenting in our clinic.

## 4 Conclusions

In summary, we present a unique case of heterotopic bone formation over the trabecular meshwork without any inciting event or predisposing condition. After biopsy confirmation, we successfully managed the patient's elevated intraocular pressure with a trabeculectomy surgery. Our report highlights the importance of early detection and timely intervention for such rare conditions to prevent irreversible vision loss.

## Patient consent

Consent to publish the case report was obtained. This report does not contain any personal information that could lead to the identification of the patient.

## Funding

No funding or grant support.

## Authorship

All authors attest that they meet the current ICMJE criteria for Authorship.

## Declaration of competing interest

The authors declare that they have no known competing financial interests or personal relationships that could have appeared to influence the work reported in this paper.
